# Orbital Pseudotumor as an Extrahepatic Complication of Chronic HCV Infection

**DOI:** 10.1155/2018/5498953

**Published:** 2018-05-30

**Authors:** Owen Huang, Tina Shah, Divya Sundarapandiyan, Matthew J. Akiyama

**Affiliations:** Montefiore Medical Center, New York, NY, USA

## Abstract

We present a patient who was admitted for eye swelling, pain, and discharge, with CT orbits with contrast demonstrating inflammation and enlargement of the lacrimal glands and surrounding tissue. He was found to have an HCV infection for unknown duration upon further investigation. All other workup (autoimmune, rheumatic, and infectious) was unrevealing. The patient was diagnosed with orbital pseudotumor as an extrahepatic complication of chronic HCV infection, and he was managed with prednisone which dramatically decreased his eye swelling and pain. Steroid treatment may serve as a bridge to suppress symptoms of extrahepatic manifestations, especially in ocular cases, while HCV-infected patients await treatment to eradicate their HCV infection.

## 1. Background

Hepatitis C virus (HCV) affects greater than 185 million people worldwide with an estimated global prevalence of 2.8 percent [1]. Following HCV infection, approximately 50 to 85 percent of cases will proceed to develop into a chronic infection [2]. Chronic infection is most notable for causing liver fibrosis and eventual cirrhosis, with estimated 20 to 25 percent of individuals with chronic HCV infection eventually developing cirrhosis over a 25- to 30-year period [3]. This further increases the likelihood of morbidity and mortality due to the increased risk for hepatocellular carcinoma, variceal hemorrhage, ascites, hepatic encephalopathy, spontaneous bacterial peritonitis, and death. In the United States, chronic HCV remains one of the most frequent causes for liver transplantation despite recent advances in HCV treatment [4]. Although the literature on HCV focuses primarily on liver pathology and consequences, extrahepatic manifestations are also an important cause of HCV-associated morbidity.

Some of the most commonly documented extrahepatic manifestations and associations include lymphoproliferative disorders, dermatologic conditions, autoimmune disorders, type II diabetes mellitus, and renal disease. Up to 74 percent of individuals with chronic HCV infection experience some form of extrahepatic manifestation [5]. Up to 80 percent of patients with essential mixed cryoglobulinemia are infected with HCV, and nearly 50 percent of patients with HCV infection have low levels of circulating mixed cryoglobulins [6, 7]. The most common manifestations of this disease include leukocytoclastic vasculitis with palpable purpura and petechiae, arthralgias, renal disease, neurologic disease, and hypocomplementemia. There is also documented association of HCV infection with lymphomas, specifically non-Hodgkin's lymphomas and specific B-cell non-Hodgkin's lymphoma subtypes (marginal zone lymphoma, diffuse large B-cell lymphoma, and lymphoplasmacytic lymphoma). A meta-analysis of 7 large studies demonstrated statistically significant detection of HCV infection in 3.6 percent of non-Hodgkin's lymphomas compared to 2.7 percent in the control group, with an odds ratio of 1.78, with even higher increased risk in the subtypes listed above [8]. Several studies have reported decreased prevalence of lymphomas in HCV-treated individuals when compared nontreated. A variety of dermatologic conditions are also associated with HCV infection, most notably sporadic porphyria cutanea tarda. A systemic review of 50 studies demonstrated that nearly 50 percent of patients with porphyria cutanea tarda had a concurrent HCV infection [9]. The disease presents with photosensitivity and skin fragility leading to erythema and vesicle formation, in addition to markedly elevated urine uroporphyrin. Other dermatologic conditions include lichen planus, necrolytic acral erythema, and as mentioned previously, leukocytoclastic vasculitis. A number of autoimmune disorders have also been found to be associated with chronic HCV infection, most commonly Sjogren syndrome, rheumatoid arthritis, and systemic lupus erythematous. Data demonstrate increased autoantibody production (such as ANA, rheumatoid factor, or smooth muscle autoantibodies) in chronically infected HCV patients. For example, ANA is present in 32 to 61 percent of patients [10, 11]. The association of diabetes mellitus with HCV infection is controversial. A meta-analysis of 34 studies estimated a 70 percent increased risk and odds ratio of 1.7 in HCV-infected individuals when compared to the control group [12]; however, HCV-infected patients also tend to have confounding comorbidities, such as older age and obesity, in addition to diabetic patients having more parenteral exposure and liver damage causing glucose intolerance. The most well-established renal pathology associated with HCV infection is membranoproliferative glomerulonephritis (commonly associated with essential mixed cryoglobulinemia). A number of studies have documented a decrease in proteinuria with treatment of HCV.

Less often discussed are ocular pathologies as a result of HCV. Current literature documents direct ocular associations that include retinopathy, keratoconjunctivitis sicca, and Mooren's ulcer. However, because HCV is also associated with autoimmune phenomena, the possibility of autoimmune conditions of the eye such as IgG4-related ocular pathologies must also be considered. Patients with IgG4-related ophthalmic disease usually involve the lacrimal gland and may cause diseases such as orbital pseudotumor or sclerosing dacryoadenitis. Recently, there has been a case report documenting the association of chronic HCV infection complicated by inflammatory dacryoadenitits and orbital pseudotumor as highly unusual extrahepatic manifestations of chronic HCV [13]. Orbital pseudotumor is an idiopathic condition of inflammation associated with many inflammatory and autoimmune conditions such as IgG4-related disease that can involve the extraocular muscles, lacrimal gland, other orbital structures and tissue.

Here, we present an unusual case of a man who presented with a swollen eye and also found to have chronic HCV infection.

## 2. Case Presentation

**Figure 1:**
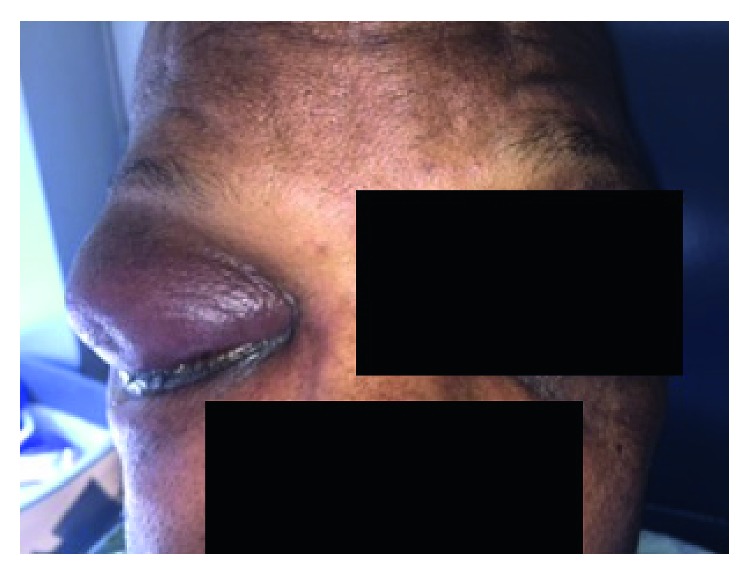
Image of the patient's eyes upon presentation, significant for swelling, ptosis, chemosis, and mucous discharge.

The patient was a 63-year-old African American male with medical history significant for tongue cancer status after surgery and radiation therapy over 5 years ago presenting with right eye pain, upper lid swelling, ptosis, significant chemosis, and mucous discharge. He reported that he had chronic swelling and discharge in his eyes bilaterally for a “long time”; however, the four days leading to presentation the pain began worsening and the swelling increased until his right eye became swollen shut (Figure 1). He did not report an inciting incident, such as trauma or change in activity, and did not attempt to self-medicate or relieve the swelling. He denied fevers or chills. On physical exam, the patient reported pain to extraocular motions and decreased vision. He had normal pupillary reaction and no neurological deficits. The rest of his physical exam was normal as well. Liver function tests were as follows: ALT 39 U/L, AST 100 U/L, total bilirubin 1.0 mg/dL, direct bilirubin 0.3 mg/dL, albumin 4.1 g/dL, INR 1.0, and platelet count 150k/*µ*L. The rest of his completed blood count (CBC) included WBC 4.2k/*µ*L, hemoglobin 10.8 g/dL, and hematocrit 31.1%.

### 2.1. Investigation

**Figure 2:**
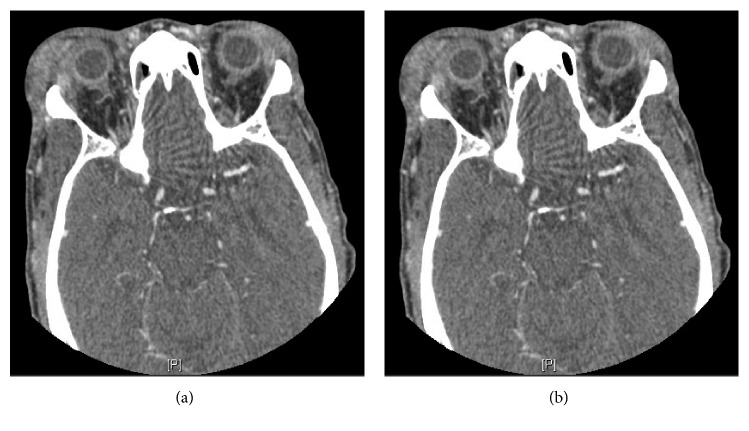
CT orbits with contrast demonstrating enlargement and inflammation within the lacrimal glands and the surrounding tissues: (b) greater than (a).

**Figure 3:**
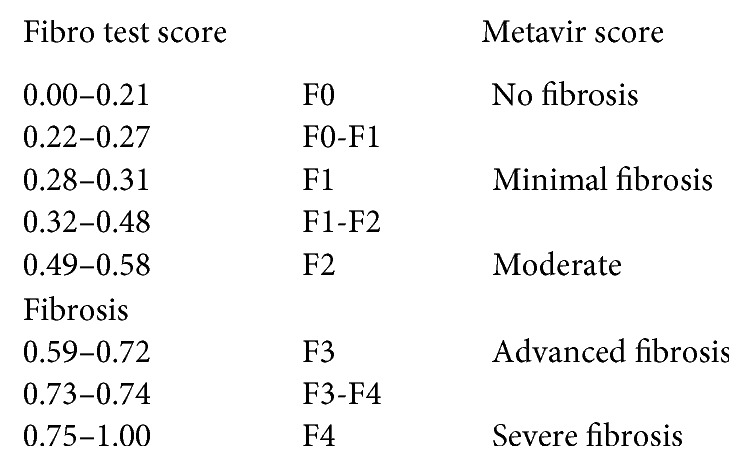
Metavir stage guide.

The patient was admitted to the general medicine floor for treatment and workup. He was sent for a computed tomography (CT) scan of the orbits with contrast, which demonstrated enlargement and inflammation within the lacrimal glands and the surrounding tissues, right greater than left (Figure 2). He received an extensive laboratory autoimmune and rheumatic workup, including antinuclear, anti-Sjogren's syndrome (SSA and SSB), antineutrophil cytoplasmic, myeloperoxidase, and proteinase 3 antibody tests, and thyroid studies (including thyroid peroxidase antibody), all of which were negative. He also received a chest X-ray (looking for hilar lymphadenopathy), which was unremarkable. CBC was also unremarkable. However, upon testing for HCV infection, the patient was found to be positive for HCV antibody, with an HCV viral load of approximately 600k. The genotype was found to be 1A. It was unclear how the patient may have contracted this infection as the patient was hesitant upon further exploration of his social history. Further infectious workup for *Treponema pallidum*, HIV, hepatitis B, hepatitis A, and Epstein–Barr virus was unrevealing. He underwent liver fibrosis testing, which revealed a fibrosis score of 0.30, demonstrating minimal liver fibrosis (F1) using the Metavir stage guide (Figure 3).

### 2.2. Differential Diagnosis and Treatment

The initial differential diagnosis was infectious versus inflammatory: orbital cellulitis versus dacryoadenitis (infectious or noninfectious) versus orbital pseudotumor versus IgG4-related disease. Given his presentation with significant swelling and discharge in his right eye and moderately in his left eye, the initial concern was for orbital cellulitis. He was treated with ampicillin/sulbactam and erythromycin ointment over two days with minimal improvement, making an infectious etiology less likely. In addition, he had no leukocytosis or fever. The bilateral findings were more suspicious for an inflammatory process; therefore, 1 g IV methylprednisone for a 3-day course was then initiated with marked improvement of his eyes in appearance, pain, and drainage, further suggesting an inflammatory process affecting the lacrimal glands and surrounding tissue (as documented by CT orbits). Inflammatory dacryoadenitis and orbital pseudotumor were diagnosed. The etiology of these inflammatory conditions were suspected to be secondary to the extrahepatic HCV phenomenon, possibly autoimmune relating to IgG4-related disease, which can arise via chronic HCV infection as an autoimmune phenomena. At discharge, the patient was prescribed prednisone 80 mg daily with a taper.

### 2.3. Outcome and Follow-Up

The patient was scheduled to have an incisional biopsy for further pathological workup but did not attend his ophthalmology appointment two days following discharge. However, he did visit his primary care provider for initiation of HCV treatment. At this visit, it was documented that the patient's eye was further improved. Additional workup was ordered for approval and initiation of HCV treatment; however, the patient was lost to follow-up after his initial visit. He was ultimately documented to have passed away soon after his initial visit due to a cardiac event.

## 3. Discussion

This is a case of a patient presenting with inflammatory dacryoadenitis and orbital pseudotumor likely as a highly unusual extrahepatic manifestation of HCV infection. Currently, lymphocytic sialadenitis with sicca symptoms suggestive of Sjogren syndrome is commonly described in patients with chronic HCV infection. A systematic review of 11 studies demonstrated that the prevalence of Sjogren-like symptoms was 12 percent among HCV-infected patients [14]. These symptoms are classically caused by lymphocytic infiltration of glands, most commonly the lacrimal and salivary glands, as a result of an autoimmune phenomenon. Orbital pseudotumor may similarly affect the lacrimal gland with histological biopsy similarly demonstrating lymphocytic infiltration. Orbital pseudotumor is defined as idiopathic; however, it is possible that there is significant overlap in cases of orbital pseudotumor and Sjogren syndrome within chronically infected HCV patients, such as this patient. It is possible that the infiltration unusually extended beyond the glandular borders into the adjacent soft tissue, as demonstrated by the CT orbits with contrast exhibiting inflammation and enlargement of both the lacrimal glands and surrounding tissue. This extraglandular inflammation likely caused the swelling and pain the patient experienced.

In patients with chronic HCV infection, eradication of the infection is a key target in the therapeutic approach of treating extrahepatic manifestations [15]. In patients presenting with unexplained eye pathology such as in this case of orbital pseudotumor, physicians should consider testing for HCV infection or at the very least assessing for risk factors for HCV infection. If successfully treated by HCV eradication, these cases of idiopathic orbital pseudotumor in the setting of HCV infection could be further stratified as HCV-associated orbital pseudotumor. In addition, for this patient, the use of steroids to decrease pain and swelling was very successful. Steroids can act as a bridge to relieve and treat symptoms while awaiting HCV treatment, as coordinating HCV treatment may be complicated by circumstances such as medical insurance, setting up appointments, and so on. Treatment of HCV should then, theoretically, eliminate the extrahepatic symptoms.

Our case report has several limitations. First, we were unable to obtain a pathologic specimen from the patient to confirm a diagnosis. Second, the patient never received HCV treatment and additionally passed away; therefore, it is unknown whether treatment of his HCV would have led to complete resolution of his eye pathology.

## Additional Points

*Learning Points*. (i) In patients with unexplained eye pathology, consider testing for HCV infection as a source of etiology as HCV is a treatable condition in light of recent advances in HCV therapy, and extrahepatic manifestations of HCV are treated by eradication of the virus. (ii) Extrahepatic manifestations of chronic HCV infections are common and should always be considered as a cause of pathology. (iii) Autoimmune phenomena, in the setting of HCV infection, may cause extrahepatic manifestations and must be considered in the differential for HCV-infected patients. (iv) Steroid treatment may serve as a bridge to suppress symptoms of extrahepatic manifestations, such as this case of eye inflammation, while HCV-infected patients await eradication of their HCV infections.
